# Supplementary material for: GWAS and fine-mapping of livability and six disease traits in Holstein cattle
Source: BMC Genomics. 2020 Jan 13;21:41. doi: 10.1186/s12864-020-6461-z (PMC6958677; doi:10.1186/s12864-020-6461-z)
Supplement: Supplementary file 1 — Additional file 1. Boxplot with PTA reliability for hypocalcemia (CALC), displaced abomasum (DSAB), ketosis (KETO), mastitis (MAST), metritis (METR), retained placenta (RETP) and cow livability. [file 12864_2020_6461_MOESM1_ESM.docx]

**Additional File 1:** Boxplot with PTA reliability for hypocalcemia (CALC), displaced abomasum (DSAB), ketosis (KETO), mastitis (MAST), metritis (METR), retained placenta (RETP) and cow livability.

| 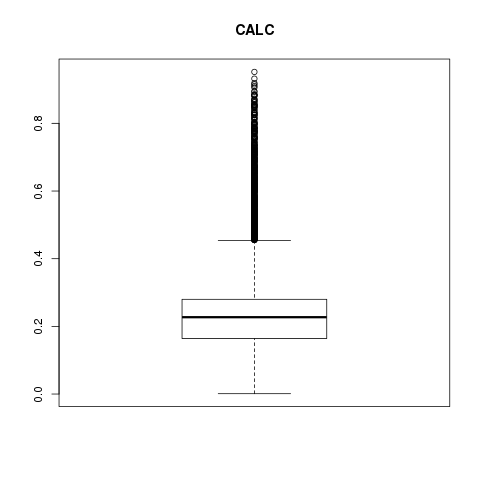 | 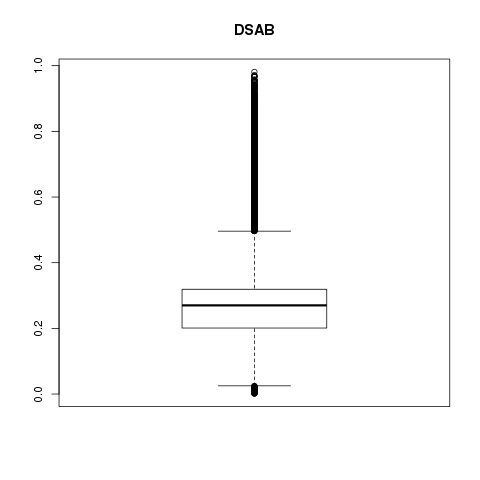 |
| --- | --- |
| 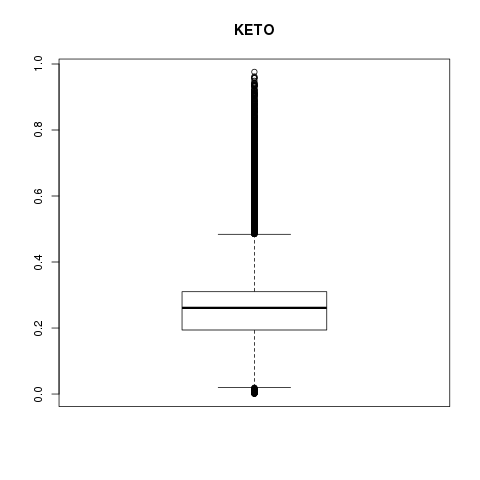 | 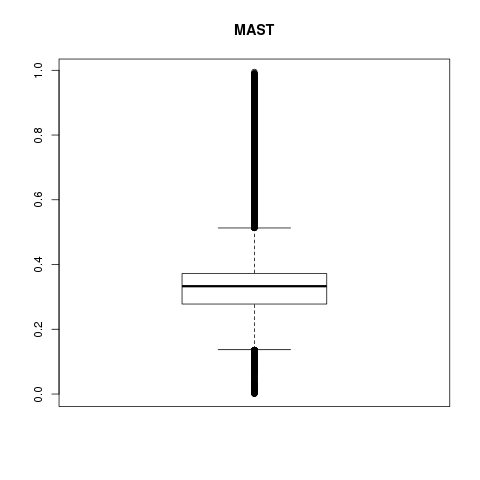 |
| 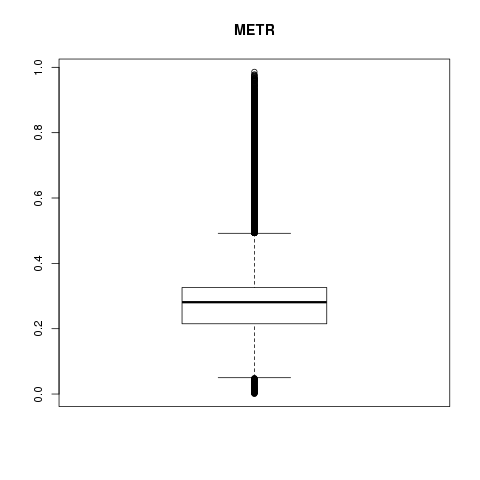 | 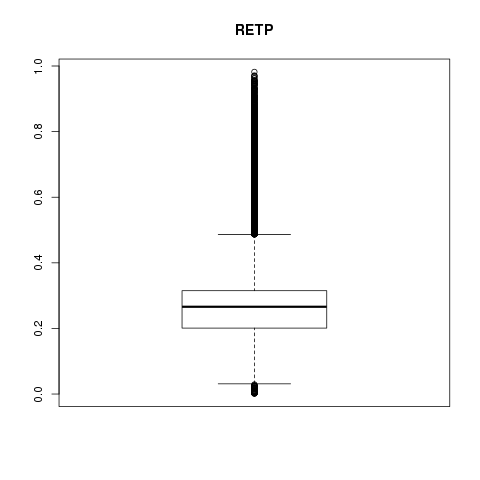 |
| 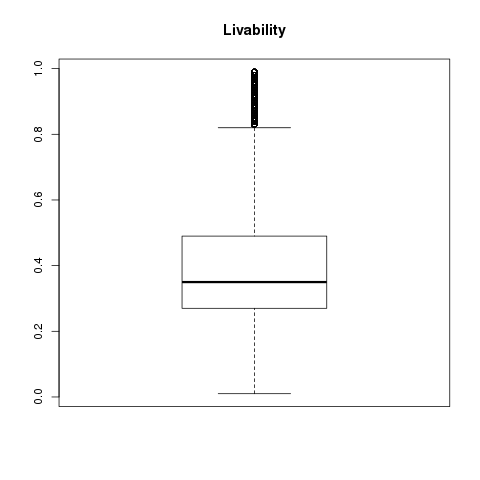 | |
