# Supplementary material for: GWAS and fine-mapping of livability and six disease traits in Holstein cattle
Source: BMC Genomics. 2020 Jan 13;21:41. doi: 10.1186/s12864-020-6461-z (PMC6958677; doi:10.1186/s12864-020-6461-z)
Supplement: Supplementary file 3 — Additional file 3. List of variants into genes with highest posterior probability of causality mostly associated with displaced abomasum (DSAB), ketosis (KETO), mastitis (MAST), metritis (METR), retained placenta (RETP) and cow livability. [file 12864_2020_6461_MOESM3_ESM.docx]

**Additional File 3:** List of variants into genes with highest posterior probability of causality mostly associated with displaced abomasum (DSAB), ketosis (KETO), mastitis (MAST), metritis (METR), retained placenta (RETP) and cow livability.

| Trait | Gene | Variant | MAF | GWAS  *P*-value | Causality  *P*-value | N_PPC | Annotation |
| --- | --- | --- | --- | --- | --- | --- | --- |
| DSAB | *PLXNA4* | 4:97101981 | 0.02 | 4.5×10^-7^ | 6.5×10^-7^ | 0.26 | Intergenic |
| DSAB | *FANCC* | 8:83052202 | 0.11 | 1.3×10^-7^ | 2.1×10^-9^ | 0.19 | Intron |
| DSAB | *FANCC* | 8:83083934 | 0.11 | 1.3×10^-7^ | 2.1×10^-9^ | 0.19 | Intron |
| DSAB | *FANCC* | 8:83123780 | 0.11 | 1.3×10^-7^ | 2.1×10^-9^ | 0.19 | Intron |
| DSAB | *NTM* | 29:35977236 | 0.07 | 1.3×10^-7^ | 1.7×10^-7^ | 0.91 | Intergenic |
|  | *LOC107133096* | 14:2762595 | 0.03 | 2.0×10^-9^ | 5.9×10^-8^ | 0.89 | Upstream/Intergenic |
|  | *PARP10* | 14:2025096 | 0.23 | 7.0×10^-7^ | 1.7×10^-5^ | 0.02 | Missense/Downstream |
| KETO |  | 14:2026646 | 0.23 | 7.0×10^-7^ | 1.7×10^-5^ | 0.02 | Upstream/Intron |
|  | *DGAT1* | 14:1802266 | 0.22 | 1.0×10^-6^ | 2.1×10^-5^ | 0.02 | Missense/Downstream |
|  |  | 14:1804647 | 0.22 | 1.0×10^-6^ | 2.1×10^-5^ | 0.02 | Downstream |
| MAST | *GC* | 6:88718227 | 0.45 | 2.0×10^-7^ | 1.2×10^-7^ | 0.13 | Intron |
| METR | *COBL* | 4:4643092 | 0.13 | 4.3×10^-3^ | 7.7×10^-4^ | 0.04 | Intron |
|  | *LOC100296627* | 4:32578298 | 0.22 | 7.6×10^-7^ | 4.0×10^-13^ | 1 | Intron |
| RETP | *MALSU1* | 4:32057434 | 0.17 | 7.5×10^-4^ | 1,1×10^-13^ | 0 | Upstream/Intergenic |
|  | *ABCB1* | 4:33063807 | 0.12 | 6.3×10^-1^ | 8.4×10^-3^ | 0 | Intron |
| RETP | *QDPR* | 6:117727506 | 0.024 | 2.2×10^-4^ | 2.3×10^-5^ | 0 | 3_prime_UTR_variant |
| RETP | *QDPR* | 6:117727635 | 0.024 | 2.2×10^-4^ | 2.3×10^-5^ | 0 | 3_prime_UTR_variant |
| RETP | *QDPR* | 6:117727851 | 0.024 | 2,2×10^-4^ | 2.3×10^-5^ | 0 | 3_prime_UTR_variant |
| RETP | *QDPR* | 6:117728023 | 0.024 | 2.2×10^-4^ | 2.3×10^-5^ | 0 | Missense |
| RETP | *QDPR* | 6:117743248 | 0.024 | 2.2×10^-4^ | 2.3×10^-5^ | 0 | Missense |
| RETP | *TMEM182* | 11:7465110 | 0.06 | 9.0×10^-8^ | 9.9×10^-8^ | 0.95 | Intron |
| RETP | *LOC783493* | 18:63802274 | 0.24 | 8.3×10^-3^ | 2.3×10^-3^ | 0 | Intron |
| Livability | *ABCC9* | 5:88823164 | 0.47 | 1.5×10^-10^ | 1.5×10^-10^ | 0.51 | Splice/Intron |
| Livability | *GC* | 6:88718227 | 0.45 | 1.9×10^-17^ | 1.4×10^-19^ | 0.03 | Intergenic |
| Livability | *ZFAT* | 14:8249314 | 0.04 | 2.1×10^-5^ | 3.0×10^-5^ | 0 | Intron |
| Livability | *LOC618463* | 18:57589121 | 0.07 | 1.7×10^-20^ | 3.1×10^-20^ | 0.19 | Intron |
| Livability | *CCDC88C* | 21:56700449 | 0.01 | 8.6×10^-11^ | 8.9×10^-11^ | 0.46 | Intron |
| Livability | *LOC101908667* | 23:25905667 | 0.02 | 2.1×10^-8^ | 7.9×10^-9^ | 0.08 | Intergenic |

N_PPC = Normalized posterior probability of causality
